# Evolution and containment of transmissible recombinant vector vaccines

**DOI:** 10.1111/eva.12806

**Published:** 2019-06-12

**Authors:** Scott L. Nuismer, Andrew Basinski, James J. Bull

**Affiliations:** ^1^ Department of Biological Sciences University of Idaho Moscow Idaho; ^2^ Department of Mathematics University of Idaho Moscow Idaho; ^3^ Department of Integrative Biology The University of Texas at Austin Austin Texas

**Keywords:** emerging infectious disease, infectious disease, one health, recombinant vector vaccine, reservoir, self‐disseminating vaccine, spillover

## Abstract

Transmissible vaccines offer a revolutionary approach for controlling infectious disease and may provide one of the few feasible methods for eliminating pathogens from inaccessible wildlife populations. Current efforts to develop transmissible vaccines use recombinant vector technology whereby pathogen antigens are engineered to be expressed from innocuous infectious viral vectors. The resulting vaccines can transmit from host to host, amplifying the number of vaccine‐protected individuals beyond those initially vaccinated directly through parenteral inoculation. One main engineering challenge is the potential for natural selection to favor vaccine mutants that eliminate or reduce expression of antigenic inserts, resulting in immunogenic decay of the vaccine over time. Here, we study a mathematical model of vector mutation whereby continuous elimination of the antigenic insert results in reversion of the vaccine back into the insert‐free vector. We use this model to quantify the maximum allowable rate of reversion that can be tolerated for a transmissible vaccine to maintain a critical threshold level of immunogenicity against a target pathogen. Our results demonstrate that even for transmissible vaccines where reversion is frequent, performance will often substantially exceed that of conventional, directly administered vaccines. Further, our results demonstrate the feasibility of designing transmissible vaccines that yield desired levels of immunogenicity, yet degrade at a rate sufficient for persistence of the recombinant vaccine within the environment to be minimized.

## 1 INTRODUCTION

The spillover of zoonotic pathogens from wild animal reservoirs into the human population is becoming an ever more frequent, but still largely unpredictable occurrence with significant negative impacts on human health (Plowright et al., 2017). Even the most predictable zoonoses, such as Lassa fever virus from *Mastomys natalensis* and rabies from feral dogs, are estimated to kill thousands of individuals per year worldwide (Richmond & Baglole, 2003; Taylor & Nel, 2015). Less predictable spillover events also create large public health impacts. For instance, the 2014 West African Ebola epidemic killed more than 11,000 people over the course of the 30‐month epidemic (Kaner & Schaack, 2016), and outbreaks of SARS and MERS have killed hundreds (de Wit, Doremalen, Falzarano, & Munster, 2016). Although large‐scale vaccination campaigns of wild animals have shown the ability to reduce spillover events in temperate regions (e.g., Europe and North America for rabies in red foxes and raccoons), similar efforts cannot provide the coverage needed for control of most emerging zoonoses because of difficulties in reaching wildlife populations with conventional vaccines in the more remote and inaccessible environments where these pathogens frequently emerge.

One promising innovation for the vaccination of wildlife is the use of transmissible, rather than individually administered, vaccines. Conventional, nontransmissible vaccines protect only those individuals directly vaccinated (e.g., administered parenterally or through bait). In contrast, transmissible vaccines have the potential to reach more of the targeted wildlife population through animal‐to‐animal transmission. A transmissible vaccine works on the same principles as other live vaccines—following administration to an individual, the vaccine leads to stimulation of a protective immune response—but with its capacity for transmission, the vaccine is able to spread and immunologically protect other susceptible individuals beyond those directly vaccinated. Results from mathematical modeling indicate that transmissible vaccines substantially increase vaccine coverage and thereby the scope for pathogen elimination or eradication (Basinski et al., 2018; Nuismer et al., 2016; Nuismer, May, Basinski, & Remien, 2018). To date, transmissible vaccines remain largely untested. However, a transmissible vaccine comprised of an attenuated myxoma virus encoding an antigen from rabbit hemorrhagic fever virus (RHFV) was able to transmit between rabbits and proved protective against both myxoma and rabbit hemorrhagic fever (Angulo & Barcena, 2007; Bárcena et al., 2000). Efforts are now underway to develop transmissible vaccines targeting other pathogens in wildlife populations including Ebola and Lassa fever viruses in their natural animal reservoirs (Marzi et al., 2016; Murphy, Redwood, & Jarvis, 2016; Tsuda et al., 2011, 2015).

Of the two most common types of live vaccines, attenuated and recombinant vectored, the latter appears to be the most promising for development as a transmissible vaccine platform (Bull, Smithson, & Nuismer, 2018; Murphy et al., 2016). Recombinant vector vaccines (RVVs) consist of an antigen‐expressing pathogen gene(s) engineered into a competent but avirulent viral vector (Bull et al., 2018). The possible combinations afforded by this approach appear almost unlimited, and the ability to choose vectors from a wide range of possibilities should permit rates and pathways of vaccine transmission to be tuned to at least some degree. However, recombinant vaccines face regulatory issues due to their genetically modified status, and the ability to transmit—a big advantage in pathogen control—may provide a hurdle to regulatory approval. There are multiple means by which transmissible RVVs may overcome potential regulatory issues for their use in wildlife populations. If the vaccine is only weakly transmissible (e.g., $R_{0}<1$), the vaccine will inevitably approach extinction once it is no longer being directly administered to the target population (Nuismer et al., 2016). Similarly, if the vector occurs naturally in the host population, vector‐specific immunity may lead to vaccine extinction (Basinski et al., 2018). Finally, if the RVV is genetically unstable and progressively evolves to delete or downregulate the inserted transgene, the vaccine may revert over time, leaving behind only the original viral vector.

Although previous work has studied the efficacy of weakly transmissible vaccines and explored how cross‐immunity between vector and vaccine can influence vaccine performance (Basinski et al., 2018; Nuismer et al., 2016), the consequences of genetic instability for transmissible vaccines have not yet been rigorously evaluated. As a result, it is currently unclear what level of instability can be tolerated for a transmissible vaccine to remain effective, or how unstable a transmissible vaccine needs to be to guarantee its ultimate extinction. This balance between immunogenicity and target antigen loss will dictate whether vaccine decay is slow enough to enable broad protection against a target pathogen but fast enough to ensure vaccine extinction in a reasonable time frame.

In the present study, we develop and analyze mathematical models and computer simulations of transmissible recombinant vector vaccines. Our models explicitly integrate genetic instability by allowing the vaccine to revert to viral vector. We consider scenarios where cross‐immunity between vector and vaccine is absent as well as scenarios where cross‐immunity is strong. We use these models to identify the amount of genetic instability that can be tolerated for a transmissible vaccine to remain effective, and to evaluate the scope for tuning genetic instability for maximal efficacy while ensuring vaccine extinction.

## 2 THE MODELS

Recombinant vector vaccines are assumed to exist in large well‐mixed populations, where mutation combined with within‐host evolution continuously causes reversion of the vaccine to the insert‐free vector at a rate, u, either through insert deletion or through downregulation of antigenic insert expression. Although we represent reversion as a simple single‐step process, in reality it depends on a complex progression of processes (e.g., mutation, selection, sampling) playing out within‐host individuals. We assume the vaccine is continuously administered directly to a fraction, σ, of newborn animals. Although this may be difficult to accomplish in many wild animal populations, it may be possible using the distribution of baits or by directly vaccinating and releasing animals reared in captivity. Our model assumes directly vaccinated animals become infected with the vaccine and recover from vaccine infection at rate, $\gamma_{V}$. Upon recovery, previously infected animals develop lifelong immunity against the target pathogen (hence against the vaccine).

**Figure 1:**
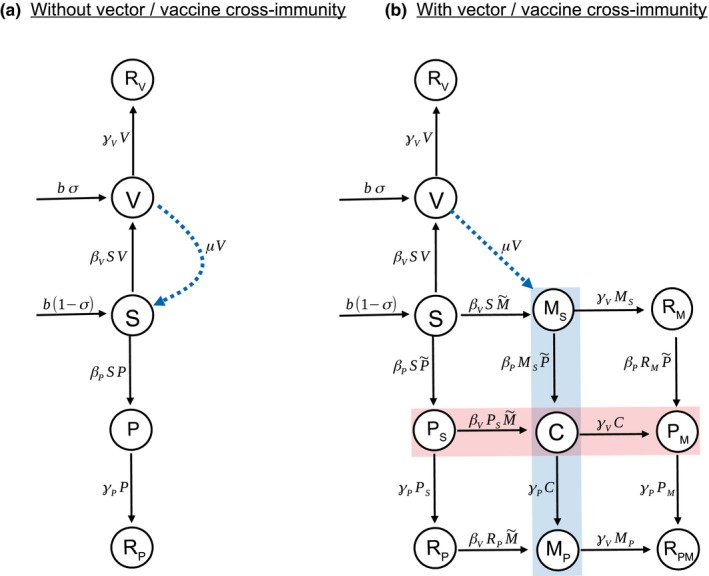
Flowcharts of the model without (a) and with (b) cross‐immunity. All classes die at rate d (not shown). In the model with cross‐immunity, shading indicates classes that are infected with pathogen (pink) and vector (blue)

The degree of cross‐immunity between viral vector and intact vaccine has been shown to influence the efficacy of recombinant vector transmissible vaccines (Basinski et al., 2018). Here, we study two models that capture the extreme ends of the cross‐immunity spectrum. In the first model, we assume a complete absence of cross‐immunity; the dynamics of vaccine and vector are independent of each other (Figure 1a). In the second model, we investigate the opposite extreme, where complete cross‐immunity between vector and vaccine exists, leading to strong competition between them for susceptible hosts (Figure 1b).

### 2.1 No cross‐immunity

An absence of cross‐immunity might accrue to a RVV if prior vaccine exposure and the resultant host adaptive immune response does not biologically impact vaccine re‐infection as reported for cytomegalovirus—(Gandhi & Khanna, 2004) or if immunity to the foreign antigen is highly immunodominant, minimizing other immunological responses to the vector. In the absence of cross‐immunity, the dynamics of the transmissible vaccine and pathogen can be described without explicitly tracking the density of vector generated by reversion: When a reversion transforms vaccine to vector, the host that was vaccine‐infected merely moves back into the pool of susceptible hosts. Consequently, we need follow only the densities of hosts susceptible to vaccine and pathogen (*S*), infected with vaccine (*V*), infected with pathogen (*P*), or recovered from vaccine infection ($R_{V}$) or pathogen infection ($R_{P}$) and immune to infection by both vaccine and pathogen. The following system of differential equations applies:

(1a) $$\frac{dS}{dt}=b\left(1-\sigma \right)-\beta_{V}SV-\beta_{P}SP-dS+\mathrm{uV}$$

(1b) $$\frac{dV}{dt}=b\sigma +\beta_{V}SV-\left(\gamma_{V}+d+u\right)V$$

(1c) $$\frac{dP}{dt}=\beta_{P}SP-\left(\gamma_{P}+d\right)P$$

(1d) $$\frac{dR_{V}}{dt}=\gamma_{V}V-dR_{V}$$

(1e) $$\frac{dR_{P}}{dt}=\gamma_{P}P-dR_{P}$$

where all parameters and variables are defined in Table 1. For simplicity, we assume immunity to the pathogen and vaccine is perfect and lifelong.

**Table 1:** Variables and parameters for the no cross‐immunity model

Parameter/variable	Description
*S*	The density of animals susceptible to vaccine, vector, and pathogen
*V*	The density of animals infected with intact vaccine
*M*	The density of animals infected with revertant vaccine
*P*	The density of animals infected with pathogen
$R_{V}$	The density of animals that have recovered from vaccine infection
$R_{P}$	The density of animals that have recovered from pathogen infection
u	The rate of reversion from vaccine to viral vector (reversions/day)
*b*	Host birth rate (births/day)
*d*	Host death rate (deaths/day)
$\gamma_{V}$	The rate of recovery from vaccine/vector infection (recoveries/day)
$\beta_{V}$	The rate of vaccine/vector transmission (transmissions infected individuals^‐1^∙day^‐1^)
$\gamma_{P}$	The rate of recovery from pathogen infection (recoveries/day)
$\beta_{P}$	The rate of pathogen transmission (transmissions∙infected individuals^‐1^∙day^‐1^)
σ	The rate at which newborn animals are vaccinated (vaccinations/day)

### 2.2 Complete cross‐immunity

Although some viral vectors are expected to elicit little if any cross‐immunity (i.e., CMV), in other cases at least some degree of cross‐immunity is likely. To bracket the range of possible scenarios, our second model assumes the opposite extreme of the first: complete cross‐immunity between vector and vaccine. Integrating cross‐immunity necessitates a more complex model where the dynamics of the vector are tracked explicitly. In this model, when a reversion transforms the vaccine into vector, the revertants are removed from the vaccine‐infected class (V) and moved to the vector‐infected class (M). Because the vector no longer carries or expresses pathogen genes, vector and pathogen share no cross‐immunity and can co‐infect. In contrast, because we assume the vaccine carries both pathogen and vector genes that elicit an immune response, cross‐immunity exists between vaccine and pathogen and vaccine and vector. We assume this cross‐immunity is complete and precludes co‐infection (Basinski et al., 2018). These assumptions give rise to the following system of differential equations:

(2a) $$\frac{dS}{dt}=b\left(1-\sigma \right)-\beta_{V}S\left(V+\tilde{M}\right)-\beta_{P}S\tilde{P}-dS$$

(2b) $$\frac{dV}{dt}=b\sigma +\beta_{V}SV-\left(\gamma_{V}+d+u\right)V$$

(2c) $$\frac{dM_{S}}{dt}=\beta_{V}S\tilde{M}-\beta_{P}\tilde{P}M_{S}+\mathrm{uV}-\left(\gamma_{V}+d\right)M_{S}$$

(2d) $$\frac{dM_{P}}{dt}=\beta_{V}R_{P}\tilde{M}+\gamma_{P}C-\left(\gamma_{V}+d\right)M_{P}$$

(2e) $$\frac{dP_{S}}{dt}=\beta_{P}S\tilde{P}-\beta_{V}P_{S}\tilde{M}-\left(\gamma_{P}+d\right)P_{S}$$

(2f) $$\frac{dP_{M}}{dt}=\beta_{P}R_{M}\tilde{P}+\gamma_{V}C-\left(\gamma_{P}+d\right)P_{M}$$

(2g) $$\frac{dC}{dt}=\beta_{V}P_{S}\tilde{M}+\beta_{P}M_{S}\tilde{P}-\left(\gamma_{V}+\gamma_{P}+d\right)C$$

(2h) $$\frac{dR_{V}}{dt}=\gamma_{V}V-dR_{V}$$

(2i) $$\frac{dR_{M}}{dt}=\gamma_{V}M_{S}-\beta_{P}R_{M}\tilde{P}-dR_{M}$$

(2j) $$\frac{dR_{P}}{dt}=\gamma_{P}P_{S}-\beta_{V}R_{P}\tilde{M}-dR_{P}$$

(2k) $$\frac{dR_{\mathrm{PM}}}{dt}=\gamma_{P}P_{M}+\gamma_{V}M_{P}-dR_{\mathrm{PM}}$$

where $\tilde{P}=P_{S}+P_{M}+C$ and $\tilde{M}=M_{P}+M_{S}+C$ are the total number of animals infected with pathogen and revertant vaccine, respectively, and all other parameters and variables are as defined in Table 2. Transmission rates and recovery rates of vector and vaccine are identical, and immunity is perfect and lifelong (as before).

**Table 2:** Variables unique to the complete cross‐immunity model

Parameter/variable	Description
*S*	The density of animals susceptible to vaccine, vector, and pathogen
*V*	The density of animals infected with intact vaccine
$M_{S}$	The density of previously “S” animals infected with revertant vaccine
$M_{P}$	The density of previously “ $R_{P}$” animals infected with revertant vaccine
$P_{S}$	The density of previously “S” animals infected with pathogen
$P_{M}$	The density of previously “ $R_{M}$” animals infected with pathogen
C	The density of animals co‐infected by revertant vaccine and pathogen
$R_{V}$	The density of animals that have recovered from vaccine infection
$R_{M}$	The density of animals recovered from revertant vaccine infection
$R_{P}$	The density of animals recovered from pathogen infection
$R_{\mathrm{PM}}$	The density of animals recovered from revertant vaccine and pathogen infection
u	The rate of reversion from vaccine to viral vector (reversions/day)
*b*	Host birth rate (births/day)
*d*	Host death rate (deaths/day)
$\gamma_{V}$	The rate of recovery from vaccine/vector infection (recoveries/day)
$\beta_{V}$	The rate of vaccine/vector transmission (transmissions infected individuals^‐1^∙day^‐1^)
$\gamma_{P}$	The rate of recovery from pathogen infection (recoveries/day)
$\beta_{P}$	The rate of pathogen transmission (transmissions∙infected individuals^‐1^∙day^‐1^)
σ	The rate at which newborn animals are vaccinated (vaccinations/day)

## 3 HOW DOES REVERSION REDUCE VACCINE COVERAGE?

We begin our analysis by identifying conditions where the level of transmissible vaccine administration precludes invasion by the pathogen and the transmissible vaccine can thus be used to protect a naïve population from a pathogen. The pathogen will be characterized by its *basic reproductive number* (denoted here as $R_{0,P}$), defined as the number of new infections generated by a single pathogen‐infected individual within an entirely susceptible population. When a recombinant vector vaccine immunizes at least a fraction $\left(1-\frac{1}{R_{0,P}}\right)$ of the host population, the population is protected from pathogen invasion. Numerical calculations suggest that these conditions are also those that result in the eradication of an endemic pathogen. Our interest is to know how much vaccine reversion can be tolerated for a transmissible vaccine to remain more effective than a conventional, nontransmissible vaccine.

### 3.1 No cross‐immunity

In the absence of cross‐immunity between vaccine and viral vector, identifying conditions where a transmissible vaccine precludes pathogen invasion is straightforward (Appendix 1). A population can be protected from pathogen invasion if the transmissible vaccine is administered to newborn individuals at a rate that exceeds the following threshold:

(3) $$\sigma_{\mathrm{crit}}=\left(1-\frac{1}{R_{0,P}}\right)\left(1-\frac{R_{0,V}}{R_{0,P}}+\frac{\mu }{d+\gamma_{V}}\right)$$

where $R_{0,V}$ is the basic reproductive number of the vaccine. This result generalizes our previous work for a transmissible vaccine that does not decay (Nuismer et al., 2016).

This threshold can be used to define the conditions under which a transmissible vaccine will outperform a standard vaccine. Specifically, dividing the right‐hand side of (3) by the vaccination rate required to achieve prophylaxis using a conventional vaccine $\left(1-\frac{1}{R_{0,P}}\right)$, yields the following expression for the relative performance of a transmissible vaccine that experiences reversion to viral vector:

(4) $$\rho =\left(1-\frac{R_{0,V}}{R_{0,P}}+\frac{\mu }{d+\gamma_{V}}\right)$$

If ρ is less than one, a transmissible vaccine will outperform a conventional, directly administered vaccine. If, in contrast, ρ is greater than one, a transmissible vaccine will perform worse than a traditional vaccine. This result seems to defy intuition—that transmission could be worse than no transmission. However, it stems simply from the assumption that individuals infected with the transmissible vaccine are allowed to decay into susceptibles (at rate μ) without first gaining immunity to the target pathogen. Thus, individuals that are directly vaccinated can “lose” the vaccination, something not allowed in the model lacking vaccine transmission. Were the comparison restricted to the same rates of decay for a nontransmissible vaccine as for a transmissible vaccine, transmission would always provide greater coverage.

**Figure 2:**
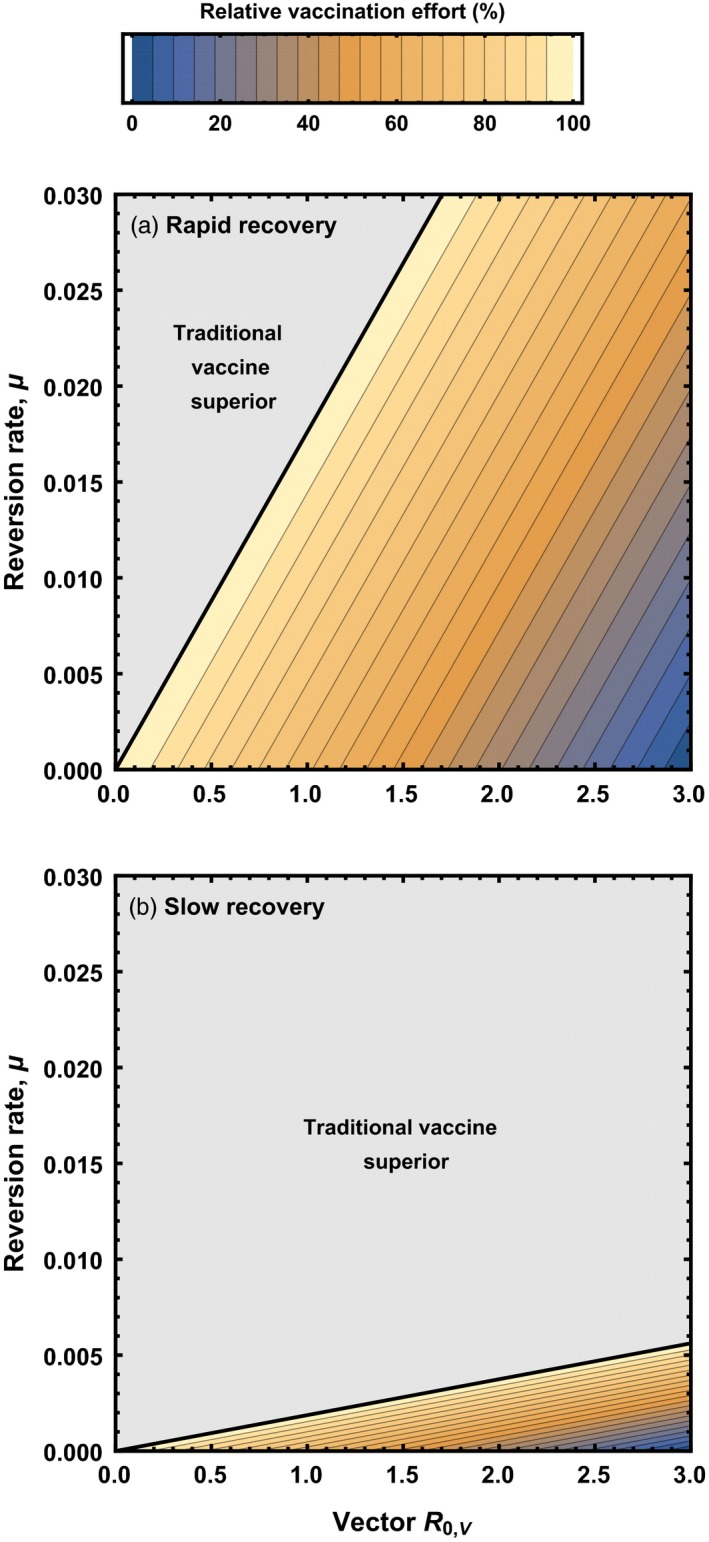
The vaccination effort (relative to a nontransmissible vaccine) required for pathogen eradication using a recombinant vector transmissible vaccine characterized by particular combinations of reversion rate to insert‐free vector (*y* axis) and vector $R_{0,V}$ (*x* axis). Cross‐immunity between vector and vaccine was assumed to be absent. The gray shaded area indicates parameter combinations for which more vaccination effort is required for a transmissible vaccine than a nontransmissible vaccine. In the top panel, the vaccine is constructed from a vector characterized by a recovery rate that is large relative to the reversion rate ($\gamma_{V}=0.05$), whereas in the bottom panel, the vector has a recovery rate that is low relative to the reversion rate ($\gamma_{V}=0.002809$). In both panels, the pathogen had an $R_{0,P}=2$, with remaining background parameters given by: b=10, d=0.002809, and $\gamma_{P}=0.05$

Inspection of (4) demonstrates that the relative performance of a transmissible vaccine depends on its rate of reversion to insert‐free vector (Figure 2). Specifically, if the reversion rate is small relative to host death rate, d, and recovery rate from vaccine/vector infection, $\gamma_{V}$, reversion has only a very slight impact on vaccine performance (Figure 2). In contrast, if the reversion rate is large relative to death and recovery rates, (4) demonstrates that reversion can have a substantial adverse effect on the performance of the transmissible vaccine (Figure 2). This implies that, all else being equal, genetically unstable transmissible vaccines that generate short‐lived infections (i.e., large recovery rate, $\gamma_{V}$) will be more effective than those that generate long‐term infections from which the host recovers only very slowly (i.e., small recovery rate, $\gamma_{V}$). The reason the rate of recovery matters, of course, is that long‐lived infections offer substantially greater scope for reversion to vector and thus may undermine the effectiveness of genetically unstable transmissible vaccines.

### 3.2 Complete cross‐immunity

**Figure 3:**
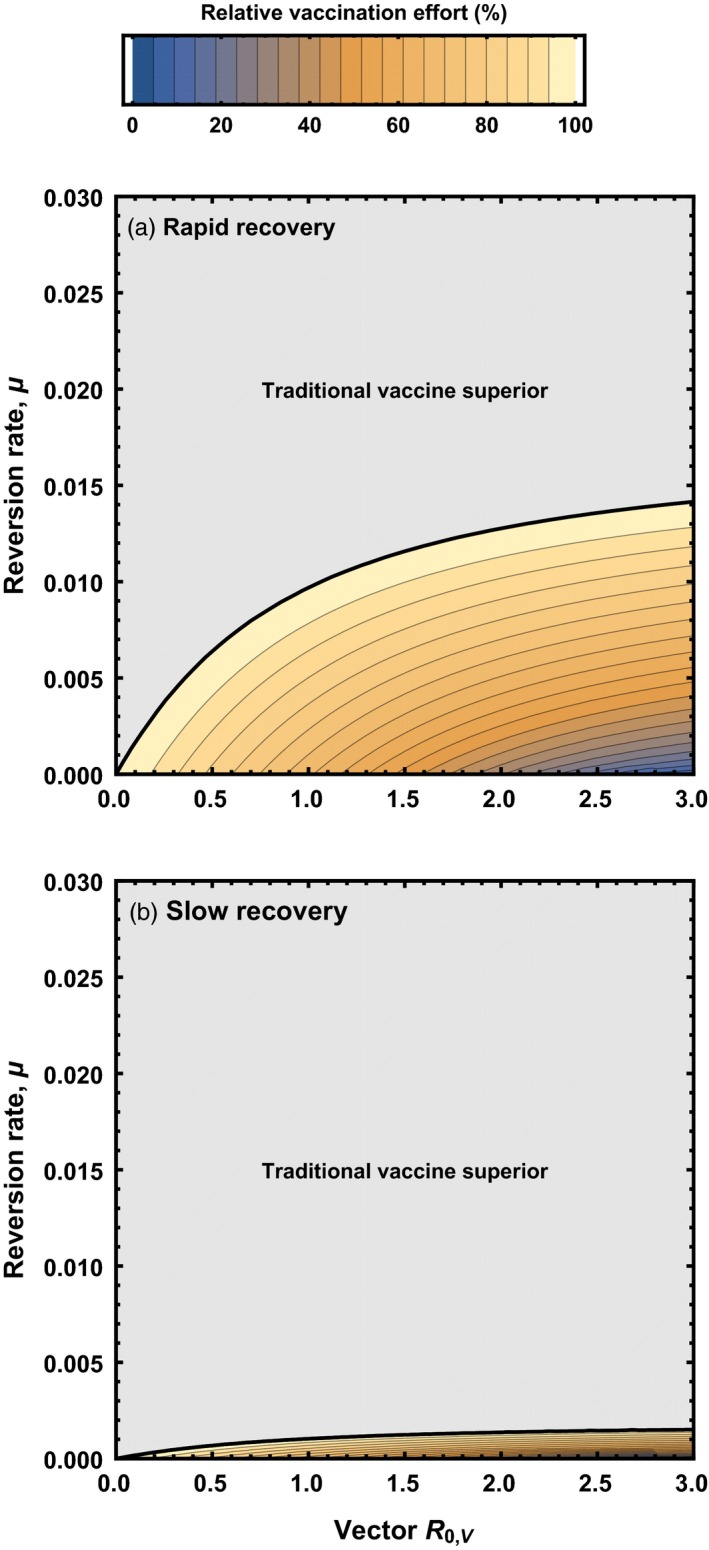
The vaccination effort (relative to a nontransmissible vaccine) required for pathogen eradication using a recombinant vector transmissible vaccine characterized by particular combinations of reversion rate to insert‐free vector (*y* axis) and vector $R_{0,V}$ (*x* axis). Cross‐immunity between vector and vaccine was assumed to be complete. The gray shaded area indicates parameter combinations for which more vaccination effort is required for a transmissible vaccine than a nontransmissible vaccine. In the top panel, the vaccine is constructed from a vector characterized by a recovery rate that is large relative to the reversion rate ($\gamma_{V}=0.05$), whereas in the bottom panel, the vector has a recovery rate that is low relative to the reversion rate ($\gamma_{V}=0.002809$). In both panels, the pathogen had an $R_{0,P}=2$, with remaining background parameters given by: b=10, d=0.002809, and $\gamma_{P}=0.05$

When cross‐immunity between vector and vaccine exists, identifying conditions under which a transmissible vaccine protects a population from pathogen invasion becomes considerably more complex (Appendix 2). Consequently, although we successfully solved for the critical level of direct vaccination required to prevent pathogen invasion, the expression becomes unwieldy. Instead, we report the key results gleaned from numerical investigations. Plotting the relative performance of a transmissible vaccine as a function of its basic reproductive number, $R_{0,V}$, and reversion rate to vector, u, demonstrates that any cross‐immunity between vector and vaccine substantially reduces the scope for a transmissible vaccine to outperform a traditional vaccine (Figure 3, compare to Figure 2). This result generalizes previous work (Basinski et al., 2018) by showing that the rate of reversion to vector plays an important role. Specifically, in the presence of cross‐immunity, even rates of reversion as low as $1.5\times 10^{-2}{day}^{-1}$ (one reversion every 66.7 days, on average), can cause a highly transmissible vaccine to be outperformed by a conventional and directly administered vaccine (Figure 3). Here too, we find that, all else being equal, transmissible vaccines with a rapid host recovery rate are more effective than those with a slow recovery rate. The benefit of rapid host recovery is, once again, the reduction in opportunities for within‐host evolution to cause reversion of genetically unstable transmissible vaccines and the production of insert‐free vector with which the vaccine must effectively compete. These results demonstrate that when cross‐immunity between vector and vaccine exists, the development of effective transmissible vaccines will depend on engineering highly stable recombinant vector vaccines with low rates of reversion to vector.

## 4 WILL THE VACCINE ULTIMATELY DEGRADE?

The foregoing results identify conditions where a transmissible vaccine outperforms a conventional, nontransmissible vaccine with respect to protecting a naïve population from pathogen invasion. Although these results identify a baseline set of conditions for determining whether a transmissible vaccine will be more effective than a standard vaccine, concerns may still exist about the ecological, evolutionary, or epidemiological consequences of releasing a potentially invasive genetically engineered virus. It is in this context that reversion from vaccine to vector may play a constructive role in containment and help to alleviate environmental concerns. Specifically, if a recombinant vector transmissible vaccine is constructed by inserting pathogen genes with antigenic activity into an endemic viral vector, mutations that delete the inserted genes transform vaccine back into vector and may ultimately return all vaccine descendants to their pre‐engineered state. Thus, mutation/reversion may enable self‐extinguishing transmissible vaccines that eradicate a pathogen, but then decay. In this section, our goal is to identify the conditions under which a recombinant vector transmissible vaccine will decay, making the idea of a “clean” or “self‐extinguishing” transmissible vaccine a real possibility.

### 4.1 No cross‐immunity

In the absence of the pathogen and without cross‐immunity between vector and vaccine, the only limit to the spread of a recombinant vector transmissible vaccine is reversion. In Appendix 3, we show that a recombinant vector transmissible vaccine will ultimately be displaced by vector anytime the following condition holds:

(5) $$u>\left(R_{0,V}-1\right)\left(d+\gamma_{V}\right)$$

**Figure 4:**
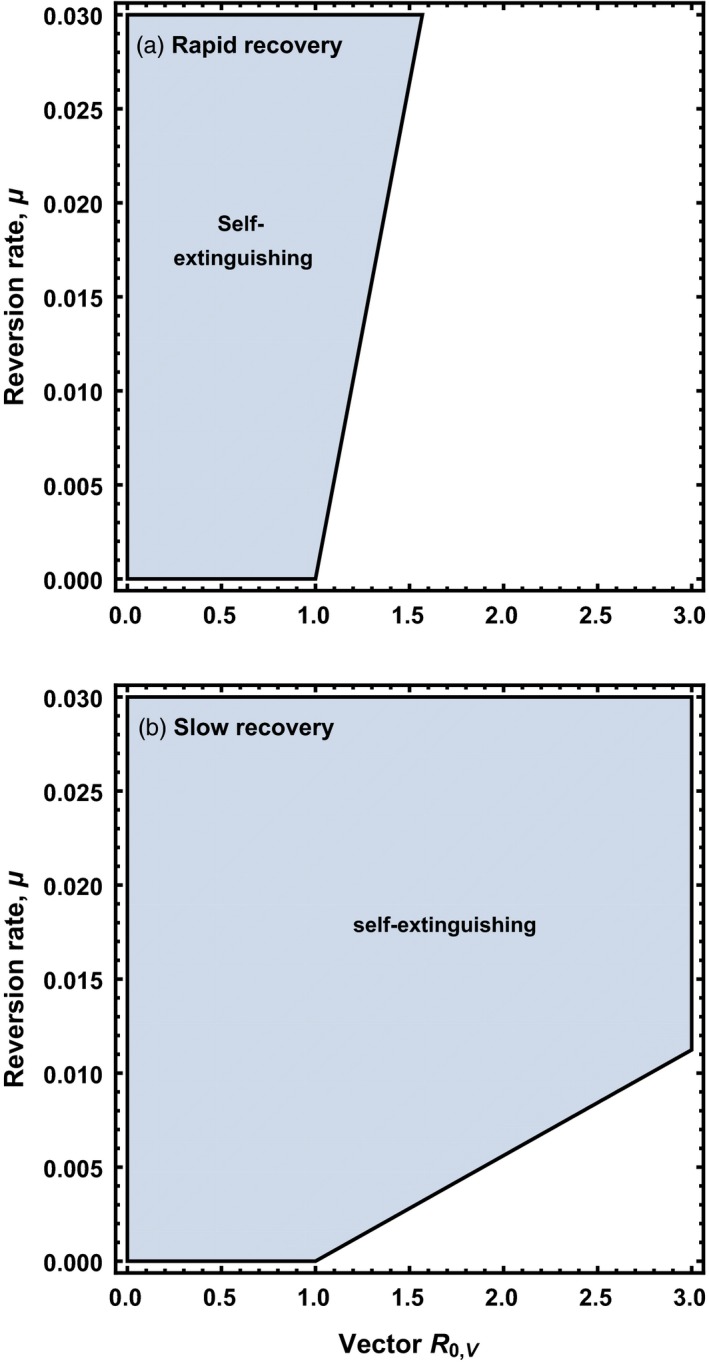
The range of reversion rates to insert‐free vector (*y* axis) and vector $R_{0,V}$ (*x* axis) for which a recombinant vector transmissible vaccine is expected to be self‐extinguishing in the absence of cross‐immunity between vector and vaccine. The gray shaded area indicates parameter combinations for which the vaccine is self‐extinguishing. In the top panel, the vaccine is constructed from a vector characterized by a recovery rate that is large relative to the reversion rate ($\gamma_{V}=0.05$), whereas in the bottom panel, the vector has a recovery rate that is low relative to the reversion rate ($\gamma_{V}=0.002809$). Remaining background parameters given by: b=10, d=0.002809, and $\gamma_{P}=0.05$

Thus, the more transmissible the vaccine (increasing values of $R_{0,V}$), the greater the rate of reversion must be for the vaccine to be self‐extinguishing (Figure 4). Because these conditions are in direct opposition to those that increase the performance of a transmissible vaccine, an effective yet self‐extinguishing transmissible vaccine must walk a tightrope between having a low enough reversion rate to be effective against the pathogen and yet a high enough reversion rate to self‐extinguish once direct vaccination ceases. Later, we will explore the scope for a transmissible vaccine to navigate this tightrope using stochastic simulations.

### 4.2 Complete cross‐immunity

In the presence of complete cross‐immunity, both reversion and competition with viral vector act in opposition to the spread of a transmissible vaccine. Consequently, results derived in Appendix 4 demonstrate that the only condition for such a vaccine to be self‐extinguishing is that reversion occurs. Thus, even though we have ignored potential costs of carrying genetic cargo, the presence of cross‐immunity guarantees that even a slight rate of reversion from vaccine to vector tips the balance in favor of vector and guarantees the ultimate extinction of the transmissible vaccine. This process may, however, be slow if rates of reversion are not appreciable.

## 5 HOW FEASIBLE IS A SELF‐EXTINGUISHING YET EFFECTIVE VACCINE?

Given the preceding results, the scope for engineering a transmissible vaccine that is highly effective, yet guaranteed to self‐extinguish once direct vaccination ceases, is unclear. Here, we use stochastic simulations to explore the scope for a transmissible vaccine to eradicate a target pathogen, but ultimately extinguish itself from the population. Specifically, we use the Gillespie algorithm (Gillespie, 1977) to simulate the systems of differential Equations ((1a), (1b), (1c), (1d), (1e), (2a), (2b), (2c), (2d), (2e), (2f), (2g), (2h), (2i), (2j), (2k)) and ((1a), (1b), (1c), (1d), (1e), (2a), (2b), (2c), (2d), (2e), (2f), (2g), (2h), (2i), (2j), (2k)) in finite populations where stochastic extinction of the pathogen and vaccine is possible. The general scenario we focus on is one where simulations are initiated with the target pathogen endemic and at equilibrium and the vector absent. After two hundred simulation days, a transmissible vaccine is introduced into the population by vaccinating a constant proportion, σ, of newborn individuals for a period of λ days. After another 2,200 days, the number of individuals infected with the pathogen or vaccine is determined and this information recorded. Simulations were run across a range of pathogen and vaccine $R_{0}$’s, vaccine reversion rates, vaccine/vector recovery rates, and for vaccination campaigns of different intensity (σ) and duration (λ).

### 5.1 No cross‐immunity

**Figure 5:**
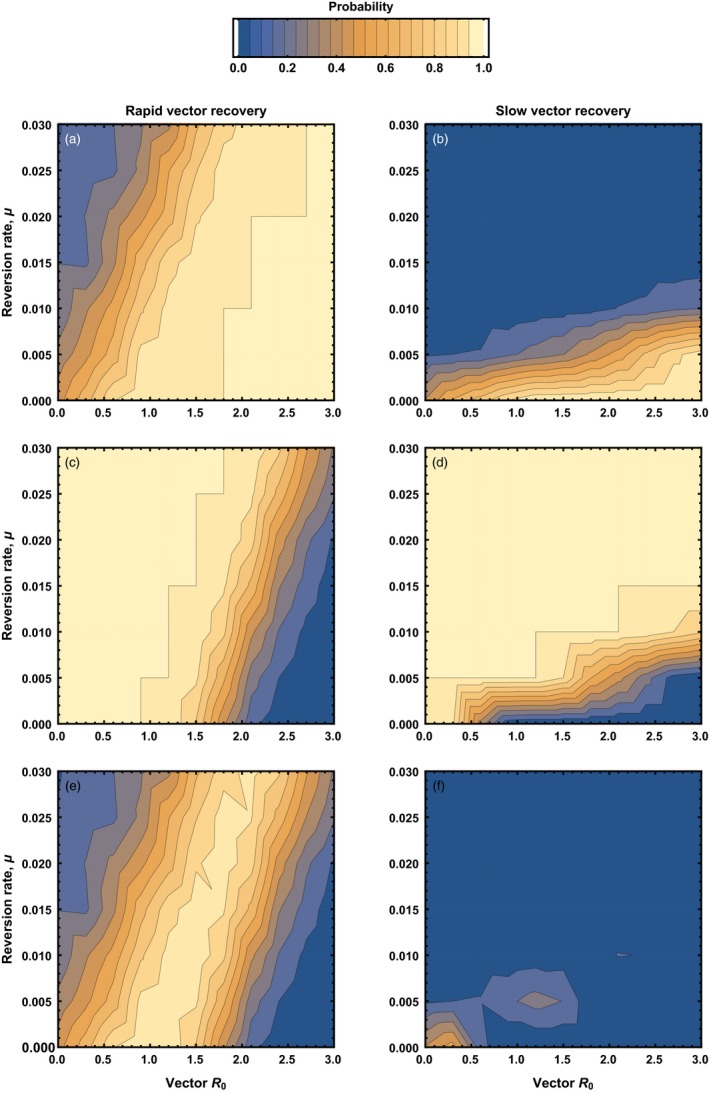
The impact of the reversion rate to insert‐free vector (*y* axis) and vector $R_{0,V}$ (*x* axis) on the probability the pathogen is eradicated (top panels), the probability that the vaccine is self‐extinguishing (middle panels), and the probability that the pathogen is eradicated, and the vaccine has been entirely degraded/extinguished (bottom panels) for the case where vector and vaccine have no cross‐immunity. In the left‐hand column, the vaccine is constructed from a vector characterized by recovery rate that is large relative to the reversion rate ($\gamma_{V}=0.05$), whereas in the right‐hand column, the vector has a recovery rate that is low relative to the reversion rate ($\gamma_{V}=0.002809$). In all panels, the pathogen had an $R_{0,P}=2$, with remaining background parameters given by: b=10, d=0.002809, and $\gamma_{P}=0.05$. In this figure, the vaccine was administered to 30% of newborn animals over a period of 720 days

In the absence of cross‐immunity, our results show that a transmissible vaccine can be an effective tool for eradicating/eliminating an endemic pathogen even when significant reversion occurs (Figure 5). At the same time, however, our results show that for such a vaccine to self‐extinguish, reversion must increase in proportion to the vaccine $R_{0,V}$. This sets up a conundrum where the better able the vaccine is to immunologically protect against the pathogen, the harder it is for the vaccine to self‐extinguish. We investigated the scope for resolving this conflict by identifying parameter combinations that allowed the vaccine to first eradicate the pathogen and then subsequently self‐extinguish (Figure 5). This analysis confirmed the challenge of engineering an effective, yet self‐extinguishing, transmissible vaccine and demonstrates that reversion rate, u, and vaccine/vector basic reproductive number, $R_{0,V}$, must fall within a narrow band of combinations (Figure 5). In addition, all else being equal, as the recovery rate ($\gamma_{V})$ increases, the range of suitable parameter combinations also increases. Thus, stochastic simulations support the deterministic results in demonstrating that developing an effective, yet self‐extinguishing, transmissible vaccine in the absence of cross‐immunity is theoretically possible but requires challenging engineering and reliable estimates of key vaccine parameters.

### 5.2 Complete cross‐immunity

**Figure 6:**
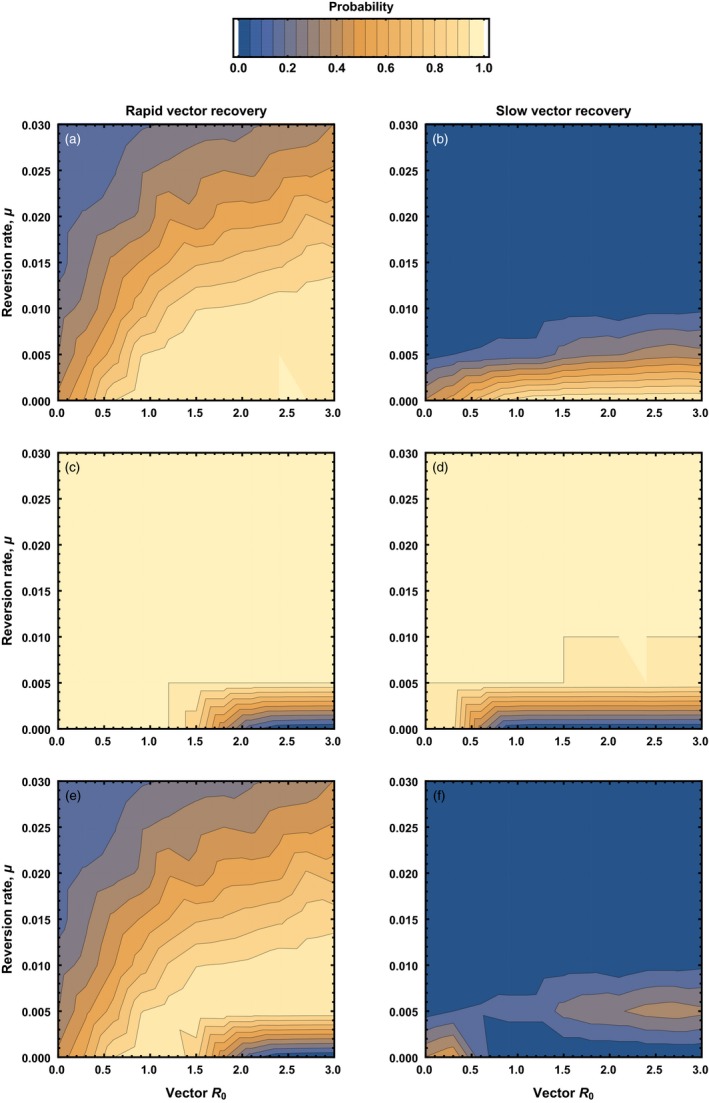
The impact of the reversion rate to insert‐free vector (*y* axis) and vector $R_{0,P}$ (*x* axis) on the probability the pathogen is eradicated (top panels), the probability that the vaccine is self‐extinguishing (middle panels), and the probability that the pathogen is eradicated, and the vaccine has been entirely degraded/extinguished (bottom panels) for the case where vector and vaccine have complete cross‐immunity. In the left‐hand column, the vaccine is constructed from a vector characterized by a recovery rate that is large relative to the reversion rate ($\gamma_{V}=0.05$), whereas in the right‐hand column, the vector has a recovery rate that is low relative to the reversion rate ($\gamma_{V}=0.002809$). In all panels, the pathogen had an $R_{0,P}=2$, with remaining background parameters given by: b=10, d=0.002809, and $\gamma_{P}=0.05$. In this figure, the vaccine was administered to 30% of newborn animals over a period of 720 days. Although the vaccine would ultimately be eliminated from the population, as predicted by our analytical results, for very low rate of reversion, this process is very slow and requires more time than our simulations were run

Stochastic simulations demonstrate that cross‐immunity between vector and vaccine reduces the scope for pathogen eradication but has little overall impact on opportunities for designing an effective and self‐extinguishing transmissible vaccine (Figure 6). The reason is that, although cross‐immunity makes pathogen eradication more challenging, it practically guarantees that a transmissible vaccine self‐extinguishes from competition with vector, although this process may be slow when rates of reversion are not appreciable. On balance, then, cross‐immunity does little to reduce opportunities for designing an effective, self‐extinguishing, vaccine. Cross‐immunity does, however, affect the parameter combinations that enable success, requiring increased transmission rates and reduced reversion rates (to see this, compare the bottom rows of Figures 5 and 6). Just as in the absence of cross‐immunity, the scope for engineering an effective and self‐extinguishing transmissible vaccine is substantially greater when recovery from vector/vaccine infection is relatively rapid (i.e., $\gamma_{V}$ is relatively large). Also, as we saw before, successful engineering will require that key parameters—such as rates of reversion and transmission—can be accurately measured and finely tuned.

## 6 ESTIMATING THE REVERSION RATE

The rate at which a recombinant vector transmissible vaccine reverts to insert‐free vector (μ) is critical for predicting both vaccine efficacy and its loss from the population when vaccination ceases. There are as yet no direct estimates of this parameter, and because it involves the interplay of multiple within‐host processes (i.e., mutation, selection, sampling), it also cannot be easily inferred from existing data on those constituent processes. What we do know, however, is that the stability of inserted transgenes, in general, varies substantially (Duch, Carrasco, Jespersen, Hansen, & Pedersen, 2004; Knowles et al., 2009; Logg, Logg, Tai, Cannon, & Kasahara, 2001; Malczyk et al., 2015; Schmerer et al., 2014; Yu et al., 2014), emphasizing the critical importance of estimating this parameter for any candidate vaccine. Here, we develop a brief description of how this parameter might be estimated. Our example is deliberately simplified and should not be considered representative of most or even many ways that such an estimation protocol might be conducted. The example is offered to provide a sense of what is involved in the estimation.

### 6.1 Experimental design

**Figure 7:**
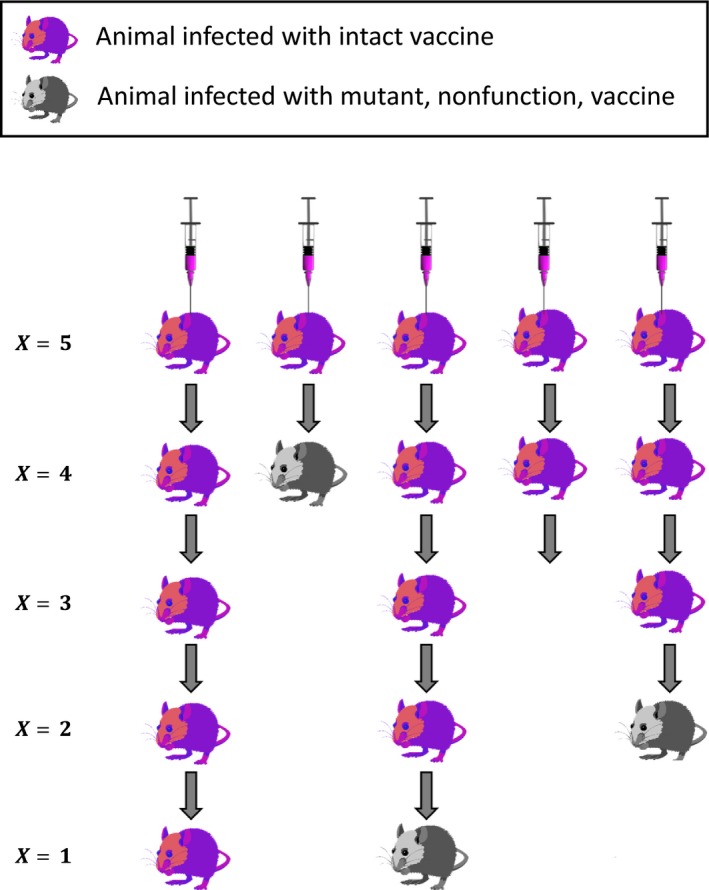
An effective experimental design for estimating the rate of reversion from vaccine to insert‐free vector. The experiment is initiated by inoculating N animals with pure vaccine. Once infection becomes established, virus is transferred to a new host animal and sequenced, and serology performed to assess immune response. Thus, the experimental design should minimize variation in transmission and guarantee that transmission occurs from one animal to the next. Viral sequence and/or serological results are used to determine the number of animals carrying intact/effective vaccine (X). Although only five replicate lineages are shown, accurate estimation of the reversion rate may require tens or even hundreds of animals, depending on the reversion rate. The experiment should be continued until some appreciable number of animals are carrying revertant vaccine, with accuracy maximized when roughly half of all animals are carrying revertant vaccine

A straightforward approach to estimating u for a recombinant vector vaccine is through replicated serial passage experiments (Figure 7). A possible design would inoculate replicate animals with intact vaccine. These generation‐0 animals would be housed (individually) with uninfected animals and their status monitored. When uninfected individuals became infected with vaccine (generation‐1), they would each be housed with a new, uninfected individual (generation‐2), who is then monitored. A line would be terminated if an uninfected individual either remained uninfected beyond the period of its partner's infectiousness or became infected with revertant only. Individuals would be scored for their infection status (also observing individuals infected with vaccine for conversion to revertant). Instead of relying on natural infection, this process could also be carried out by direct transfer of virus from the infected to the uninfected individual, as suggested in the figure, although such a design faces many decisions that could affect the rate of reversion (e.g., when to manually transmit, how much virus to transmit, which tissues to inoculate).

### 6.2 Parameter estimation

The experimental design presented above allows the rate of reversion to be estimated using maximum likelihood. We note that any known design could be subjected to its own likelihood analysis, and ours was chosen merely to keep the illustration tractable. The data required are the fraction of the initial lines still carrying vaccine at each point in time, adjusted to exclude lines in which no transmission occurred. This approach neglects issues such as viral polymorphism (or dichotomizes it). The probability that a lineage carries intact vaccine at time *t* in the experiment is given by:

(6) $$P_{V}=Exp\left[-\mathrm{ut}\right]$$

where *t* is the number of days since the experiment began and all individuals were infected with intact vaccine at the start of the experiment. The probability of observing X lineages with vaccine given a total of *N* replicate lineages is then given by:

(7) $$L=\left(\begin{array}{c} N\\ X \end{array}\right){P_{V}}^{X}{\left(1-P_{V}\right)}^{N-X}$$

and the maximum likelihood solution for the reversion rate is given by:

(8) $$\hat{u}=\frac{Log[\frac{n}{X}]}{t}$$

If PCR and/or serological tests have been conducted at multiple times over the course of the experiment, more refined estimates of the reversion rate can be achieved by integrating this information. Specifically, if $N_{1}$, $N_{2}$, …, $N_{\tau }$ lineages have been assayed on days, $t_{1}$, $t_{2}$, … $t_{\tau }$, and $X_{1}$, $X_{2}$, …, $X_{\tau }$ of these lineages found to be PCR and/or serologically positive for the insert, the likelihood function is:

(9) $$L=\prod_{i=1}^{\tau }\left(\begin{array}{c} N_{i}\\ X_{i} \end{array}\right){P_{V}}^{X_{i}}{\left(1-P_{V}\right)}^{N_{i}-X_{i}}$$

Numerically maximizing this function, or the equivalent log‐likelihood expression, with respect to μ yields an estimate for the key reversion rate parameter of our model.

## 7 DISCUSSION

Our goal has been to investigate the consequences of transmissible vaccine instability on vaccine efficacy and fate. We have focused on recombinant vector vaccines, for which instability is the loss or downregulation of an antigenic insert and loss of vaccine function. Our results demonstrate that recombinant vector vaccines can remain effective, even when genetically unstable and prone to reversion to insert‐free vector. Not surprisingly, this is particularly true for transmissible vaccines that experience little cross‐immunity with the viral vector. In addition to confirming the importance of cross‐immunity, our results show that, all else being equal, those vaccines that transmit rapidly and generate only short‐lived infection will be the most tolerant to genetic instability. Short‐lived infections are favorable because they reduce opportunities for mutation and strain selection within hosts to increase the abundance of revertant vaccine.

In addition to demonstrating that transmissible vaccines can be robust to genetic instability, our results suggest that it may be possible to capitalize on this instability to develop self‐extinguishing transmissible vaccines. The most immediate reason for ensuring that transmissible vaccines self‐extinguish is for regulatory reasons. In this respect, deletion of the insert would restore the vaccine to the status of viral vector, an entity that may be naturally circulating in the wild population. Fortunately, deletion is also the process most easily controlled by engineering. For example, deletions are well known to occur between direct repeats in DNA (Cunningham et al., 1997; Springman, Molineux, Duong, Bull, & Bull, 2012), and modification or elimination of those repeats reduces the spontaneous rate of deletions (Sleight, Bartley, Lieviant, & Sauro, 2010). Genome engineering may also afford the possibility of designing accelerating rates of genomic instability. Our model assumed a constant rate of decay to revertant; a rate that accelerated over time would have many advantages. Such methods are not obviously ready for implementation, but they are at least suggested by “counting mechanisms” that have been employed in bacteria (reviewed in Bull & Barrick, 2017).

Our study contributes to an increasing awareness of the many issues facing the choice of an appropriate vector for a transmissible recombinant vector vaccine. In most cases, the choice of a vector may be based on considerations that lie outside model results, and the model results will then inform the possibilities and avenues for success. Consider cytomegalovirus (CMV), a vector currently being used in the development of transmissible vaccines targeting Ebola and Lassa fever (Marzi et al., 2016; Murphy et al., 2016; Tsuda et al., 2011, 2015). CMV appears promising because of its high level of species specificity, and ability to readily reinfect previously infected hosts (Murphy et al., 2016), a property shown to be critically important here, and in a previous study (Basinski et al., 2018). In contrast, our results also illuminate a potential weakness of CMV, which tends to generate long‐lived infections that our results suggest increase opportunities for vaccine reversion. Ultimately, a rigorous assessment of vector quality will require robust estimates for key parameters such as transmission rates and rates of reversion to vector, something that should be a central focus of ongoing efforts to engineer successful transmissible vaccines.

As with any model, ours make simplifying assumptions that may influence our conclusions. One important assumption is that reversion from vaccine to vector has no impact on rates of transmission, despite the clear reasons to expect greater rates of vector transmission after the genetic cargo has been unloaded. Although we did not explicitly model this scenario, we expect doing so would support our current results. For instance, if the vector transmits more rapidly than the vaccine, we expect cross‐immunity to become even more important and the efficacy of a transmissible vaccine to be further reduced. As a consequence, the difference between vectors that generate cross‐immunity and those that do not would become even more significant. Another important assumption of our model is that it is possible to directly vaccinate newborn animals and that these newborn animals have the same rate of transmission as adult animals. If recovery from vaccine infection is rapid and newborn animals have limited encounters with the population at large, this could substantially limit the efficacy of a transmissible vaccine. Evaluating how this important assumption influences our results will require more complex models that explicitly integrate age structure. Finally, our modeling framework focuses on a single well‐mixed population and thus ignores potential impacts of spatial heterogeneity (Basinski, Nuismer, & Remien, 2019) and population structure (Varrelman, Basinski, Remien, & Nuismer, 2019).

As a whole, our results suggest transmissible vaccines may be relatively robust to modest levels of genetic instability, particularly in cases where cross‐immunity between vector and vaccine is weak. It may also prove possible to capitalize on genetic instability to develop transmissible vaccines that are both effective and self‐extinguishing. Identifying the quantitative impact transmissible vaccines are likely to have on real pathogen populations, however, will require considerable progress in estimating key parameters such as vector transmission rates, recovery rates, and rates of reversion to insert‐free vector. Only when reliable estimates become available for these parameters will it become possible to critically evaluate the promise of various vectors and the scope for designing effective and self‐extinguishing vaccines.

## CONFLICT OF INTEREST

None declared.

## Data Availability

Data sharing not applicable—no new data generated. All simulation code is available upon request.

## APPENDIX 1

### The critical vaccination threshold in the absence of cross‐immunity

We identify the vaccination rate that, in the absence of any cross‐immunity between vector and vaccine, preempts the invasion of a pathogen. If the host population is at steady state and a small number of pathogen‐infected hosts are introduced into the population, the pathogen is unable to invade the population when

(A1-1) $$\dot{P}=\beta_{p}S^{*}P-\left(\gamma_{p}+d\right)P<0.$$

Here, $S^{*}$ is the steady state density of susceptible hosts, and P is the initial (small) density of pathogen‐infected hosts. Because *p* > 0, inequality (A1‐1) can be rearranged as

(A1-2) $$S^{*}<\frac{b}{d}\frac{1}{R_{0,P}},$$

where $R_{0,p}$ is the pathogen's basic reproduction number. Inequality A1‐2 shows that the host population is protected from pathogen invasion so long as the density of susceptible hosts at steady state is below the threshold $\frac{b}{d}\frac{1}{R_{0,p}}$. Next, we find the proportion σ of newborns that must be vaccinated to maintain the susceptible population at this prophylactic threshold.

In the absence of the pathogen, the densities of susceptible, vaccine‐infected, and vaccine‐recovered hosts are described by a subset of system (1) with the P state variable set to 0, and the equations for pathogen‐infected and pathogen‐recovered hosts ($P,R_{P}$) omitted. The submodel consists of Equations 1a, 1b, and 1d:

(A1-3a) $$\frac{dS}{dt}=b\left(1-\sigma \right)-\beta_{V}SV-dS+\mathrm{uV}$$

(A1-3b) $$\frac{dV}{dt}=b\sigma +\beta_{V}SV-\left(\gamma_{V}+d+u\right)V$$

(A1-3c) $$\frac{dR_{V}}{dt}=\gamma_{V}V-dR_{V}$$

In the supplementary Mathematica file (available on request), we show that system (A1-3a), (A1-3b), (A1-3c) possesses a single biologically relevant (i.e., positive state variables) equilibrium that is locally stable.

As a result, if continual vaccination is carried out in perpetuity, the densities of susceptible, vaccine‐infected, and vaccine‐recovered hosts each approach steady state values described by this equilibrium. In particular, the density of susceptible individuals approaches

(A1-4) $$S^{*}=\frac{b\beta_{V}+d\left(d+\mu +\gamma_{V}\right)-\sqrt{4bd\sigma \beta_{V}\left(d+\gamma_{V}\right)+{\left(b\beta_{V}-d\left(d+u+\gamma_{V}\right)\right)}^{2}}}{2d\beta_{V}},$$

which was found by setting the derivatives in system (A1-3a), (A1-3b), (A1-3c) to 0 and solving for S, V, and $R_{V}$. The steady state values $V^{*}$ and ${R_{V}}^{*}$ are contained in the Mathematica file, but are omitted here.

We derive the threshold vaccination proportion by plugging (A1‐4) into the threshold condition for prophylaxis, inequality A1‐2, and solving for σ. The critical proportion is

$$\sigma_{crit}=\left(1-\frac{1}{R_{0,P}}\right)\left(\left(1-\frac{R_{0,V}}{R_{0,P}}\right)+\frac{u}{d+\gamma_{V}}\right).$$

## APPENDIX 2

### The critical vaccination threshold in the presence of cross‐immunity

Cross‐immunity between the vector and vaccine will hinder the spread of the vaccine. Consequently, a greater proportion of the newborns will have to be vaccinated to prevent pathogen invasion. In Appendix 1, we show that the population is protected from pathogen spread when the density of hosts that are susceptible to the pathogen is brought below the critical threshold, $\frac{b}{d}\frac{1}{R_{0,p}}$. We use a similar threshold condition to assess whether the population is protected; however, we must broaden the definition of susceptible hosts to include all host types that can transmit the pathogen. Specifically, we define a new state variable, $\tilde{S}$, that tracks the *effective density* of hosts that are susceptible to the pathogen. $\tilde{S}$ is the summed density of susceptible hosts (*S*), hosts that are infected with the mutant vaccine ($M_{S}$), and hosts that have recovered from infection with the mutant vaccine ($R_{M}$):

(A2-1) $$\tilde{S}=S+M_{S}+R_{M}.$$

The total rate at which an invading pathogen spreads is described by the summed rates of spread in the pathogen‐infected classes. In the Mathematica supplementary, we show that, if the population is at steady state, the total rate at which an invading pathogen spreads is

(A2-2) $$\frac{d\tilde{P}}{dt}=\beta_{p}{\tilde{S}}^{*}\tilde{P}-\left(\gamma_{p}+d\right)\tilde{P},$$

where $\tilde{P}=P_{S}+P_{M}+C$ and ${\tilde{S}}^{*}$ denotes the steady state level of $\tilde{S}$. The pathogen will not be able to invade the population so long as equation A2‐2 is <0 for an initial (small) $\tilde{P}$, which is the case so long as

(A2-3) $${\tilde{S}}^{*}<\frac{b}{d}\frac{1}{R_{0,P}}.$$

Inequality A2‐3 implies that prophylaxis is achieved when the sum of the steady state densities of S, $M_{S}$, and $R_{M}$ is below $\frac{b}{d}\frac{1}{R_{0,P}}.$ Next, we find the values of these state variables at steady state and determine the proportion of newborns that must be vaccinated to preemptively bring the density of ${\tilde{S}}^{*}$ below threshold A2‐3.

In the absence of the pathogen, the levels of vector‐infected, vaccine‐infected, and susceptible hosts are described by a simplified version of System 2 with all instances of pathogen‐infected and pathogen‐recovered state variables set to 0 (*P* = 0, *C* = 0, *R_P_* = 0, *M_P_* = 0), and their associated equations omitted. Because the recovered classes *R_V_* and *R_M_* do not influence the steady state level of ${\tilde{S}}^{*}$, they can be omitted from the analysis that follows. The reduced system is comprised of Equations 2a, 2b, 2c, and 2i of the main text:

(A2-4a) $$\frac{dS}{dt}=b\left(1-\sigma \right)-\beta_{V}S\left(V+M_{S}\right)-dS$$

(A2-4b) $$\frac{dV}{dt}=b\sigma +\beta_{V}SV-\left(\gamma_{V}+d+u\right)V$$

(A2-4c) $$\frac{dM_{S}}{dt}=\beta_{V}SM_{S}+\mathrm{uV}-\left(\gamma_{V}+d\right)M_{S}$$

(A2-4d) $$\frac{dR_{M}}{dt}=\gamma_{V}M_{S}-dR_{M}$$

The steady states of System A2 are obtained by setting the left‐hand sides to 0, and solving the resulting system of equations for S, *M_S_*, and *R_M_*. The expressions that result are omitted here due to their complexity but presented in the supplementary Mathematica file. We use the resulting steady states to calculate the effective density of susceptible hosts, ${\tilde{S}}^{*}$, and then solve for the critical value of σ that brings ${\tilde{S}}^{*}$ below the threshold that prevents pathogen invasion, inequality A2‐3. The formula that results is complex and omitted here, but displayed in the Mathematica file.

## APPENDIX 3

### Conditions for a self‐extinguishing vaccine in the absence of cross‐immunity

After using a transmissible vaccine to block an invading pathogen, it may be desirable for the vaccine infection process to self‐extinguish once direct vaccination stops. We show that this behavior is possible if the rate at which the vaccine reverts into the vector is sufficiently large. Upon cessation of direct vaccination, the levels of susceptible, vaccine‐infected, and vaccine‐recovered hosts are described by Equations 1a, 1b, and 1d of the main text, with the P state variable and vaccination parameter σ both set to 0:

(A3-1a) $$\frac{dS}{dt}=b-\beta_{V}SV-dS+\mathrm{uV}$$

(A3-1b) $$\frac{dV}{dt}=\beta_{V}SV-\left(\gamma_{V}+d+u\right)V$$

(A3-1c) $$\frac{dR_{V}}{dt}=\gamma_{V}V-dR_{V}$$

To be self‐extinguishing, we require that any solution with non‐negative initial conditions approaches the equilibrium for which only susceptible individuals are present. In the supplementary Mathematica file, we show that this occurs when

(A3-2) $$\frac{b\beta_{V}}{d(d+u+\gamma_{V})}<1.$$

If inequality A3‐2 is satisfied, the susceptible‐only equilibrium is the only biologically relevant equilibrium (i.e., all state variables are non‐negative) and, in addition, is locally stable if condition A3‐2 is satisfied, but not otherwise. As a result, condition A3‐2 is necessary for solutions of system (A3-1a), (A3-1b), (A3-1c) to approach an equilibrium with zero vaccine‐infected hosts.

We use the relationship $R_{0,V}=\frac{\beta_{V}b}{d(d+\gamma_{V})}$ to rewrite condition A3‐2 in terms of the vector $R_{0,V}$. Condition A3‐2 can be rearranged to describe a threshold rate of reversion for a self‐extinguishing vaccine:

$$\left(R_{0,V}-1\right)\left(d+\gamma_{V}\right)<\mu$$

## APPENDIX 4

### Conditions for a self‐extinguishing vaccine in the presence of cross‐immunity

We check for conditions for which, following pathogen eradication, the levels of vaccine‐infected hosts fall to zero when vaccination ceases. We use a subset of System 2, which describes the scenario in which the vaccine and vector share cross‐immunity. Because the pathogen is not present in this scenario, we only work with Equation 2a, 2b, 2c, and 2i of the main text:

(A4-1a) $$\frac{dS}{dt}=b-\beta_{V}S\left(V+M_{S}\right)-dS$$

(A4-1b) $$\frac{dV}{dt}=\beta_{V}SV-\left(\gamma_{V}+d+u\right)V$$

(A4-1c) $$\frac{dM_{S}}{dt}=\beta_{V}SM_{S}+\mathrm{uV}-\left(\gamma_{V}+d\right)M_{S}$$

(A4-1d) $$\frac{dR_{M}}{dt}=\gamma_{V}M_{S}-dR_{M}$$

System A4 has two equilibria: in the first, the vector and vaccine infection levels are zero, and the population consists entirely of susceptible hosts; in the second equilibrium, the vector transmits sufficiently to maintain positive levels of vector‐infected hosts, while the vaccine does not. Because there are no equilibria of System A4 that describe a host population with positive levels of vaccine‐infected hosts, we can conclude that levels of vaccine infection are not maintained by a stable equilibrium, nor are maintained by periodic orbits about a steady state. Barring the existence of chaotic dynamics that sustain the level of vaccine‐infected hosts, we conclude that the vaccine is unable to persist in the population. Our numerical investigations support this conclusion and do not demonstrate chaotic dynamics over the parameter simulated in this manuscript.
